# Mesenchymal Stromal Cell Derived Extracellular Vesicles Reduce Hypoxia-Ischaemia Induced Perinatal Brain Injury

**DOI:** 10.3389/fphys.2019.00282

**Published:** 2019-03-19

**Authors:** Claudia Sisa, Sharad Kholia, Jordan Naylor, Maria Beatriz Herrera Sanchez, Stefania Bruno, Maria Chiara Deregibus, Giovanni Camussi, Jameel M. Inal, Sigrun Lange, Mariya Hristova

**Affiliations:** ^1^Perinatal Brain Protection and Repair Group, EGA Institute for Women’s Health, University College London, London, United Kingdom; ^2^Department of Medical Sciences, University of Turin, Turin, Italy; ^3^2i3T, Incubator and Technology Transfer, Molecular Biotechnology Center, University of Turin, Turin, Italy; ^4^Extracellular Vesicle Research Unit and Bioscience Research Group, School of Life and Medical Sciences, University of Hertfordshire, Hatfield, United Kingdom; ^5^Tissue Architecture and Regeneration Research Group, School of Life Sciences, University of Westminster, London, United Kingdom

**Keywords:** hypoxia, ischaemia, extracellular vesicles, mesenchymal stromal/stem cells, microglia, neuroprotection

## Abstract

**Background:**

Neonatal hypoxic-ischemic (HI) insult is a leading cause of disability and death in newborns, with therapeutic hypothermia being the only currently available clinical intervention. Thus there is a great need for adjunct and novel treatments for enhanced or alternative post-HI neuroprotection. Extracellular vesicles (EVs) derived from mesenchymal stromal/stem cells (MSCs) have recently been shown to exhibit regenerative effects in various injury models. Here we present findings showing neuroprotective effects of MSC-derived EVs in the Rice–Vannucci model of severe HI-induced neonatal brain insult.

**Methods:**

Mesenchymal stromal/stem cell-derived EVs were applied intranasally immediately post HI-insult and behavioral outcomes were observed 48 h following MSC-EV treatment, as assessed by negative geotaxis. Brains were thereafter excised and assessed for changes in glial responses, cell death, and neuronal loss as markers of damage at 48 h post HI-insult.

**Results:**

Brains of the MSC-EV treated group showed a significant decrease in microglial activation, cell death, and percentage tissue volume loss in multiple brain regions, compared to the control-treated groups. Furthermore, negative geotaxis test showed improved behavioral outcomes at 48 h following MSC-EV treatment.

**Conclusion:**

Our findings highlight the clinical potential of using MSC-derived EVs following neonatal hypoxia-ischaemia.

## Introduction

Neonatal hypoxic-ischaemic (HI) brain injury is a major contributing factor to cerebral palsy and other neurological disabilities and is estimated to occur in 3 in 1000 live births in the Western world and even at higher frequency in less developed countries (Perlman, 2006; Kurinczuk et al., 2010). Oxygen deprivation has been identified as a major cause of brain injury both in term and preterm babies (Wu and Colford, 2000; Hagberg et al., 2002; Mallard et al., 2003). While 15–20% of affected neonates will die postnatally, it has been found that in surviving babies 5–10% will develop persistent motor deficiencies. Furthermore, 20–50% will suffer from sensory or cognitive abnormalities; for example approximately 15% of cerebral palsy cases are due to severe neonatal HI (Hack et al., 1992; Vohr et al., 2000; Volpe, 2012; Lee et al., 2013).

Following HI insult, changes in cellular transcription, mitochondrial function, *de novo* protein synthesis and post-translational modifications all play pivotal roles (Culman et al., 2007; Pirianov et al., 2007; Yi et al., 2007). In experimental murine models of HI, implications have for example been shown for epigenetic mechanisms (Kumral et al., 2012; Lange et al., 2014), pH changes (Kendall et al., 2011b; Uria-Avellanal and Robertson, 2014), cytokines (Kendall et al., 2011a), transcription factors (Hristova et al., 2016), and protein kinases (Thei et al., 2018). Reperfusion injury and associated reactive oxygen species, together with persistent inflammation, are also a significant contributor to brain damage in neonates following HI insult (Rocha-Ferreira and Hristova, 2016). Following positive outcomes in clinical studies of hypothermia (Wyatt et al., 2007), cooling brain or whole body to 33°C (moderate hypothermia) is currently the only strategy with a demonstrated clinical benefit. In full-term infants with moderate to severe HI, cooling significantly improves survival and disability by 11% (Shankaran et al., 2012; Jacobs et al., 2013; Azzopardi et al., 2014). Importantly, as cooling is not always effective, with 40% of treated infants still suffering neurodevelopmental disabilities, recent studies have underscored the importance of combining hypothermia with safe adjunct therapies for more substantial neuroprotection. In addition, as hypothermia equipment is not readily available worldwide, novel safe and easy to apply treatments are of pivotal importance.

Mesenchymal stromal/stem cells (MSCs) have gained popularity over the years for their potential to implement regenerative medical treatment to damaged tissues of the body (Uccelli et al., 2008; Bruno et al., 2009; Gatti et al., 2011; Bruno et al., 2012; Lee et al., 2012; Tan et al., 2014; Collino et al., 2015; Heldring et al., 2015). Stem cells participate in the maintenance of homeostasis and restoration of tissues after injury through secretion of soluble factors and extracellular vesicles (EVs). EVs (exosomes and microvesicles) are 30–1000 nm lipid bilayer-enclosed structures which are released from parental cells and participate in cell-to-cell signaling processes. EVs have been shown to transport various biologically active molecules such as proteins, mRNAs, miRNAs, lncrRNAs, DNA, and lipids to target cells (Inal et al., 2012; Yeo et al., 2013; György et al., 2015; Bruno et al., 2017; Tricarico et al., 2017; van Niel et al., 2018). These molecules can act on various cell types by signaling proliferative and/or regenerative pathways including angiogenesis, cell proliferation, and immune tolerance (Deregibus et al., 2007; Camussi et al., 2010; Ranghino et al., 2012; de Jong et al., 2014; Robbins and Morelli, 2014; Lamichhane et al., 2015; Kholia et al., 2016; Merino-González et al., 2016; Zhang et al., 2016). Anti-inflammatory factors are one key group of molecules released by MSCs that are important in mediating repair and include interleukin 1 receptor agonist (IL-1ra) (Prockop and Oh, 2012), interleukin 10 (IL-10) (Németh et al., 2009), TNF-α-stimulated gene-6 (TSG6) (Drago et al., 2013; English, 2013; Madrigal et al., 2014), prostaglandin (PG) E2 (Prockop and Oh, 2012; Drago et al., 2013; English, 2013; Németh et al., 2009), and indoleamine 2,3-dioxygenase (IDO) (Meisel et al., 2004; Croitoru-Lamoury et al., 2011; Kang et al., 2012; Lin et al., 2012; Rong et al., 2012).

Recent studies have suggested that EVs released from MSCs may be more important than the actual stem cells themselves in mediating tissue-protective effects due to the therapeutic factors secreted by EVs, including anti-inflammatory mediators, cytokines and growth factors, as well as microRNAs (Collino et al., 2015; Vallabhaneni et al., 2015; Bruno et al., 2017).

Studies in regenerative models, using total EVs or separate applications of either 100K EVs (population enriched with exosomes) or 10K EVs (population enriched with microvesicles), have led to indicate that 100K EVs provide the bulk of pro-regenerative effects, although when total EVs were used it was found that 10K EVs do not interfere negatively with 100K EVs function (Bruno et al., 2017). Other studies have found that the application of total EVs was more effective than using isolated vesicle populations of 100K EVs or 10K EVs, respectively (Wen et al., 2016). Variations in these findings have not been fully explained yet but may possibly be due to differing target tissues.

HI studies using MSCs as putative treatment, have found that these stem cells have neuroprotective potential (van Velthoven et al., 2010; Kim et al., 2012; Donega et al., 2014; Park et al., 2015; Ahn et al., 2016; Corcelli et al., 2018) and the therapeutic time window was shown to be further extended when combining MSC treatment with hypothermia (Ahn et al., 2018). Furthermore, a potential for using stem cell derived EVs was recently shown in an ovine HI study, using intravenous administration of MSC-derived EVs following transient umbilical cord occlusion *in utero*, which lead to improved brain function following MSC-EV treatment (Ophelders et al., 2016).

As intranasal administration has shown to be more efficient compared to intravenous administration in experimental HI models, due to direct transport to the CNS and bypassing peripheral elimination (Merkus and van den Berg, 2007; Hanson and Frey, 2008), we thus aimed in the present study at assessing whether intranasal administration of MSC-EVs following HI-insult would have neuroprotective effects. We used the Rice-Vannucci neonatal mouse model, which involves permanent unilateral common carotid artery occlusion followed by severe (1 h) hypoxia (Vannucci and Vannucci, 1997).

Here we provide evidence that compared to control-treated littermates, the brains of animals treated intranasally with MSC-derived EVs, immediately following HI insult, show significant neuroprotection and reduced neuroinflammation, as well as improved behavioral outcome, when assessed at 48 h post-treatment.

## Materials and Methods

### Animals and Procedures

All animal experiments and care protocols were approved by the UK Home Office (PPL 70/8784) and UCL Animal Welfare and Ethical Review Board, carried out according to the United Kingdom Animals (Scientific Procedures) Act 1986. The ARRIVE guidelines were followed. Operations were performed at post-natal day 9 (P9) on C57/BI6 mice (Charles River, United Kingdom), bred in house, using a modification of the Rice–Vannucci model of neonatal HI as previously described (Hristova et al., 2010; Lange et al., 2014; Rocha-Ferreira et al., 2018). The parental animals were bred in an environment providing 12 h light/dark cycle and food and water *ad libitum*. The HI procedure was carried out as follows: P9 mice (males and females) were anesthetized using isoflurane (5% induction, 1.5% maintenance). Permanent occlusion of the left common carotid artery was established with 8/0 polypropylene sutures followed by wound closure with tissue glue. The pups were recovered at 36°C and then were returned to the dam for 2 h, whereafter they were placed in a 36°C hypoxic chamber for 60 min, in humidified 10% oxygen/90% nitrogen at 3 L/min. Immediately following hypoxic exposure, the animals were randomly allocated to HI, EV- or PBS-treatment group. The experimental (EV) group (*n* = 15) received one intranasal dose of 6 μL of EVs (100KTOT-EV; 1.25 × 10ˆ9 particles/dose) in PBS, obtained from human bone marrow derived MSCs (Poietics hMSC, Cat no: PT-2501, Lot no: 0000446319, Lonza, Switzerland) and prepared according to Bruno et al. (2017). In brief, total EVs were isolated from characterized MSC cultures (Bruno et al., 2009), which were positive for the typical MSC markers (CD105, CD29, CD73, CD44, and CD90). The total EVs were collected from the supernatants of MSCs, cultured in fetal calf serum (FCS)-depleted RPMI medium, overnight. The EVs were a pool of 5 EV isolations with each isolation comprised of 10 flasks with a passage number ranging from P3 to P5 progressively. Cell debris and apoptotic bodies were removed by centrifugation at 3000 *g* for 20 min and thereafter total EVs were isolated by ultracentrifugation for 2 h at 100,000 *g* at 4°C and stored at -80°C until used. Details on EV characterization by nanoparticle tracking analysis (NTA, Nanosight, Malvern, United Kingdom), electron microscopy, FACS analysis and Western blotting for MSC-EV specific markers are shown in Supplementary Figure S1. EV payload characterization of proteomic and genomic content of these EVs was previously published in Bruno et al. (2017). The control treated groups received either 6 μL intranasal phosphate-buffered saline (PBS) (*n* = 14; vehicle only group) or no intervention (*n* = 16; HI only group). The pups where returned to the dam and left for 48 h before behavioral assessment and subsequent histological analysis of extracted brains. All animals (both males and females) from all litters were used in the experiments. Following the HI protocol the mums and pups were observed and scored for welfare of neonatal rodents (Lloyd et al., 2000). All pups were taken care of by the mums and achieved overall scores 0–1.

Post-natal day 9 mice were used in this particular experiment as they most closely resemble term neonates (Mallard and Vexler, 2015) and also possess injury responses phenotypically similar to white and gray matter damage, such as tissue loss, microglia-mediated immunity, cell-death-mediated apoptosis, astrogliosis, and neurobehavioral alterations (Vannucci and Vannucci, 1997). The hypoxic chamber conditions were 10% oxygen/90% nitrogen according to previously described protocols (Rocha-Ferreira et al., 2018). Differences between male and female mice were not taken into account and male and female pups were combined in the analysis, as testosterone levels in mice are similar in both genders at this age (P9–P11) (Clarkson and Herbison, 2016).

### Tissue Sample Preparation

For histological assessment, animals were sacrificed 48 h post-HI insult with an intraperitoneal injection of pentobarbitone and perfusion with 30 mL of 4% paraformaldehyde in PBS. Brains were extracted and fixed in 4% paraformaldehyde in PBS at 4°C for 1 h, followed by cryoprotection in 30% sucrose dissolved in phosphate buffer (PB) for 24 h. Thereafter brains were frozen on dry ice, sectioned by cryostat into sequential 40 μm sections and stored at -80°C until histological analysis.

### Histological Analysis

Coronal brain sections were compared between animals receiving MSC-derived EVs and control-treated animals (PBS-vehicle or HI only), for microglial activation, cell death, and infarct size. Tissue sections were scored blindly by two independent observers.

### Microglial Activation Assessment

Tissue staining was performed according to established protocols as previously described (Hristova et al., 2010; Lange et al., 2014; Rocha-Ferreira et al., 2018). In brief, cryosections were thawed and rehydrated in distilled water, spread onto glass slides coated with 0.5% gelatine under a dissecting microscope, dried for 10 min, fixed in 4% formaldehyde in 100 mM PB for 5 min, treated with acetone (50, 100, 50%: 2 min each), 0.1% bovine serum albumin (PB/BSA) and washed twice in PB. The sections were pre-incubated with 5% goat serum (Sigma, St. Louis, MO, United States) in PB for 30 min and incubated overnight at 4°C with an antibody against αMβ2 integrin (Serotec, Oxford, United Kingdom) 1/5000. The sections were then washed in PB/BSA, PB, PB, PB/BSA (2 min each), incubated with biotin-labeled anti-rat IgG (Vector Laboratories, Inc., Burlingame, CA, United States) and visualized with Avidin-Biotinylated peroxidase Complex (ABC, Vector Laboratories, Inc.) and diaminobenzidine/hydrogen peroxide (DAB) stain. Sections were processed through alcohol and xylene and mounted with DEPEX (Sigma). Per animal, five cryosections (400 μm apart) of each brain were stained. Semi-quantitative scores for microglial activation were obtained as follows: 0 = no activation; 1 = focal activation; 2 = mild phagocytic activation affecting <50% of the region, thus showing diffuse activation with occasional phagocytic macrophages; 3 = phagocytic activation affecting >50% of the region, thus showing widespread activation with predominant phagocytic macrophages; 4 = total phagocytic activation (Rocha-Ferreira et al., 2015, 2018; Hristova et al., 2016). Microglial activation was scored for the following brain regions: cortex, pyriform cortex, hippocampus, striatum, thalamus, and external capsule. These regions were selected based on known selective vulnerability as a result of increased metabolic rate (Martin et al., 1997; Barkovich et al., 1998; Johnston et al., 2001, 2002; Volpe, 2001; McQuillen et al., 2003; Castillo, 2007; Leisman et al., 2014; Schmidt-Kastner, 2015).

### TUNEL Staining

For assessment of changes in cell death, brain tissue sections were stained at 400 μm intervals for DNA fragmentation using terminal deoxynucleotidyl transferase dUTP nick end labeling (TUNEL) according to the manufacturer’s instructions (Roche, United Kingdom). Cell death was quantified by averaging the numerical count of TUNEL-positive nuclei in three representative optical fields in each brain region, comparing EV-treated versus control-treated brains.

### Infarct Volume Measurement

Infarct volume was measured by Nissl stain in five coronal sections at 400 μm intervals from each forebrain, stained with cresyl violet (VWR, United Kingdom). The Optimas 6.5 imaging analysis software (Bothell, WA, United States) was used to calculate the surviving brain tissue in each brain region as percentage between experimental and control side to estimate reduction in infarct size following EV treatment compared to control groups, according to previously described methods (Kendall et al., 2006).

### Behavioral Assessment by Negative Geotaxis

The negative geotaxis test, assessing the labyrinthine reflex which reflects the strength and co-ordination of neonatal mice, was used for behavioral analysis in EV treated and control-treated or non-treated groups at 48 h post insult, before sacrificing the animals for brain extraction and subsequent histological assessment. The order of testing of the animals within the litters was randomly assigned. The mice were placed in the center of a 45° incline board with their heads facing downwards. The time for the animal to make a 180° turn to face upward and begin to move up the hill was recorded. Time was capped at 30 s. If the animal failed to complete the task by this time, 30 s was the recorded outcome. Each animal attempted this task three times and these values were averaged per animal (Rocha-Ferreira et al., 2018). The animal numbers tested per group were as follows: MSC-EV treated: *n* = 10; PBS-control treated: *n* = 10; and HI-untreated control: *n* = 13.

### Statistical Analysis

Statistical analysis was carried out with GraphPad Prism 7 (La Jolla, CA, United States), using brain region as the repeated measure for the following statistical analyses: The same six forebrain regions (cortex, pyriform cortex, hippocampus, striatum, thalamus, external capsule) were used for each outcome and each assay. Only the TUNEL data-set passed the D’Agostino and Pearson normality test. Normalized data (only one data set: TUNEL-thalamus) were analyzed using the one-way ANOVA test and the Tukey’s multiple comparisons test.

It is likely that with repeated measures such as these the observations from a single subject are correlated, therefore the first stage of the analysis included the observations from all the regions tested in a single mixed model with a random subject effect, to produce an estimate of the treatment effect and associated inference that accounts for the correlations in the data arising from the repeated measures. Further *post hoc* Tukey’s multiple comparisons test was carried out to assess evidence for subregional differences, *p* < 0.05. Other regional data sets, being non-normalized data, were analyzed using the Kruskal–Wallis test and the Dunn’s multiple comparisons test. For each outcome, the overall effect from the mixed linear model is reported, followed by the results from the individual regional *post hoc* tests. All data is presented as means and standard error of the mean (SEM) for each group. Group sizes (*n* = 15 EV, *n* = 14 PBS, *n* = 16 HI), were based on power calculations for test power >0.80 and significance <0.05. Statistical significance was determined for *p*-value <0.05.

Statistical significance in the negative geotaxis behavioral testing was assessed through Kruskal–Wallis with Dunn’s multiple comparison test, as the data was non-normally distributed. Normal distribution was assessed using Shapiro–Wilk normality test. The data is presented as individual values and median ± interquartile bars.

## Results

### Intranasal Application of MSC-Derived EVs Decreases Microglial Activation in HI

**Figure 1:**
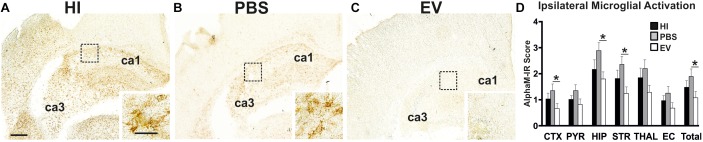
Intranasal application of MSC-derived EVs, immediately following HI, significantly reduces microglial activation after 48 h. **(A–C)** Ipsilateral overview of early microglial activation in EV- and control treated animals. Note the strong microglial activation in the HI **(A)** and PBS-treated **(B)** groups with αM+ cells showing phagocytic morphology at high magnification (**A,B** – inserts, hippocampus), compared to the EV-treated brains exhibiting a ramified phenotype (**C** – insert). The ca1 and ca3 areas of hippocampus are indicated for reference. **(D)** Intranasal EV treatment significantly reduced ipsilateral microglial activation score (**D**, Mean + SEM) overall compared to control-treated groups (PBS or HI) (Kruskal–Wallis test, *p* = 0.0168; Dunn’s test EV vs. PBS ^∗^*p* = 0.0175) with significant individual decrease in cortex (Kruskal–Wallis test, *p* = 0.0371; Dunn’s test EV vs. PBS ^∗^*p* = 0.0319), hippocampus (Kruskal–Wallis test, p = 0.0451; Dunn’s test EV vs. PBS ^∗^*p* = 0.0392), striatum (Kruskal–Wallis test, *p* = 0.0442; Dunn’s test EV vs. PBS ^∗^*p* = 0.0376). CTX, cerebral iscocortex; PYR, pyriform cortex; HIP, hippocampus; STR, striatum, Thal, thalamus; EC, external capsule. Scale bars: **A–C** = 150 μm; inserts = 15 μm.

Microglial activation (AlphaM), as assessed through αMβ2 integrin immunoreactivity, was significantly reduced in the EV-treated, compared to control PBS-treated groups overall (Kruskal–Wallis test *p* = 0.0168, Dunn’s multiple comparison test *p* = 0.0175) with subregional differences in cortex (*p* = 0.0319), hippocampus (*p* = 0.0392), and striatum (*p* = 0.0376) (Figure 1). Only a trend toward reduction, but not reaching significance, was observed in the other brain regions (Figure 1). Combining all six regions, using the Kruskal–Wallis test, the mean AlphaM scores varied significantly between treatment groups (*p* = 0.0168) (Figure 1).

### Intranasal Application of MSC-Derived EVs Decreases HI-Mediated Cell Death

**Figure 2:**
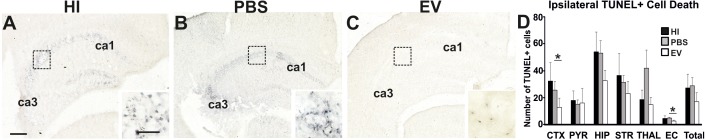
Intranasal application of MSC-derived EVs, immediately following HI, significantly reduces TUNEL+ cell death after 48 h. **(A–C)** Histochemical overview of TUNEL+ cell death in the ipsilateral forebrain of HI **(A)**, PBS- **(B)**, and EV-treated **(C)** animals. Note the typical pyknotic nuclear morphology of the TUNEL+ cells observed in the HI and PBS groups (**A,B** – inserts, hippocampus) and the lack of such cells in the EV-treated group (**C** – insert). The ca1 and ca3 areas of hippocampus are indicated for reference. **(D)** Quantification of the number of TUNEL+ cells per 20× eye-field (Mean + SEM). Compared to HI and PBS-treated control animals, Intranasal EV treatment significantly reduced ipsilateral TUNEL+ cell death in cortex (Kruskal–Wallis test, *p* = 0.0305; Dunn’s test EV vs. PBS ^∗^*p* = 0.0320) and external capsule (Kruskal–Wallis test, *p* = 0.0131; Dunn’s test EV vs. PBS ^∗^*p* = 0.0117). CTX, cerebral iscocortex; PYR, pyriform cortex; HIP, hippocampus; STR, striatum; Thal, thalamus; EC, external capsule. Scale bars: **A–C** = 150 μm; inserts = 15 μm.

Intranasal application of MSC-derived EVs significantly reduced TUNEL+ cell death in cortex and external capsule (Figure 2). Using the Kruskal–Wallis test, the mean ipsilateral TUNEL+ cell counts per representative area in the cortex varied significantly between treatment groups (EV: 12.86; PBS: 26.09; HI: 32.82; *p* = 0.0305). Using the Dunn’s multiple comparisons test, significant decrease was found in EV-treated compared to the PBS treated control group (*p* = 0.0320).

The mean ipsilateral TUNEL+ cell counts per representative area in the external capsule varied significantly between treatment groups (Kruskal–Wallis test, EV: 2.125; PBS: 4.556; HI: 4.09; *p* = 0.0131). Dunn’s multiple comparisons test showed significant decrease in the TUNEL+ cell counts in the EV-treated compared to control PBS-treated group (*p* = 0.0117) (Figure 2).

### Intranasal Application of MSC-Derived EVs Decreases HI-Mediated Volume Loss

**Figure 3:**
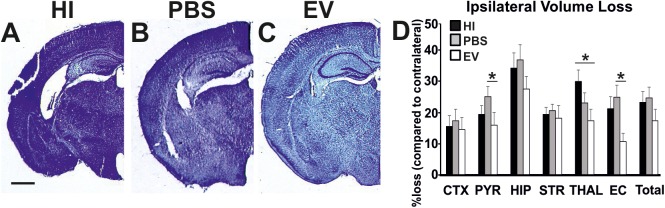
Intranasal application of MSC-derived EVs, immediately following HI, significantly reduces tissue loss after 48 h. Ipsilateral forebrain Nissl staining (Cresyl-Violet, at rostral parietal level) of HI **(A)**, PBS- **(B)**, and EV-treated **(C)** animals and quantification of ipsilateral brain tissue volume loss **(D)** at 48 h following HI-insult. Intranasal EV treatment reduced volume loss (Mean + SEM) compared to HI and PBS-treated littermates (Kruskal–Wallis test, *p* = 0.0453) with significant, individual decrease in pyriform cortex (Kruskal–Wallis test, *p* = 0.0273; Dunn’s test EV vs. PBS ^∗^*p* = 0.0219), thalamus (Kruskal–Wallis test, *p* = 0.0445; Dunn’s test EV vs. HI ^∗^*p* = 0.0348), and external capsule (Kruskal–Wallis test, *p* = 0.0114; Dunn’s test EV vs. PBS ^∗^*p* = 0.0126). CTX, cerebral iscocortex; PYR, pyriform cortex; HIP, hippocampus; STR, striatum; Thal, thalamus; EC, external capsule. Scale bars: **A–C** = 470 μm.

Intranasal EV-treatment resulted in significant reduction of tissue loss as assessed through NISSL staining, compared to control treated littermates overall (Kruskal–Wallis test, *p* = 0.0453), as well as in pyriform cortex, thalamus, and external capsule (Figure 3).

The mean ipsilateral volume loss in the pyriform cortex varied significantly between treatment groups (Kruskal–Wallis test, EV: 16.13%; PBS: 25.5%; HI: 19.63%; *p* = 0.0273). Dunn’s multiple comparisons test showed significant decrease in the EV-treated compared to the PBS-treated groups (*p* = 0.0219). The mean ipsilateral volume loss in the thalamus varied significantly between treatment groups (Kruskal–Wallis test, EV: 17.53%; PBS: 23.43%; HI: 30.44%; *p* = 0.0445). Dunn’s multiple comparisons test showed significant reduction in the EV-treated compared to HI groups (*p* = 0.0348). The mean ipsilateral volume loss in the external capsule varied significantly between treatment groups (Kruskal–Wallis test, EV: 10.8%; PBS: 25.21%; HI: 21.63%; *p* = 0.0114). Dunn’s multiple comparisons test showed significant decrease in the EV-treated compared to the PBS-treated control groups (*p* = 0.0126) (Figure 3).

### Intranasal Application of MSC-Derived EVs Improves Behavioral Outcomes

**Figure 4:**
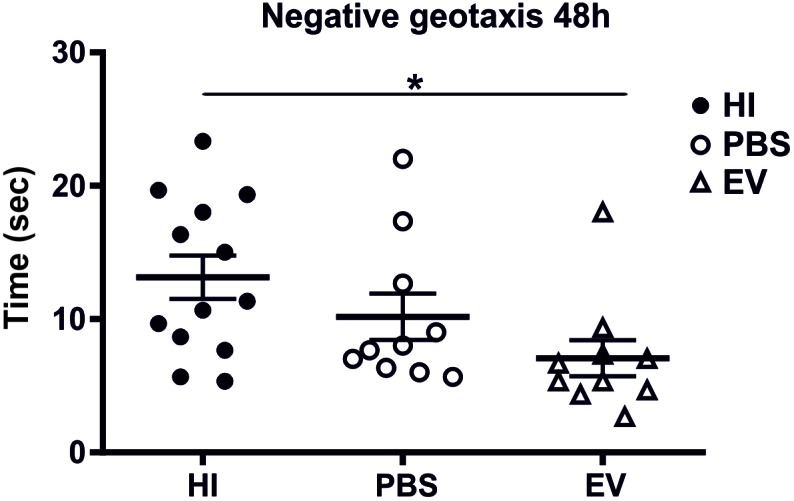
Intranasal application of MSC-derived EVs, immediately following HI reduces the time required to change orientation in negative geotaxis following neonatal HI at 48 h (postnatal day 11). Intranasal treatment with MSC-derived EVs immediately following HI significantly reduces the time necessary for change of orientation compared to HI and PBS-treated littermate controls (Kruskal–Wallis test, *p* = 0.0151, Dunn’s test EV vs. HI ^∗^*p* = 0.0114).

Intranasal administration of MSC-derived EVs significantly improved behavioral outcomes using the negative geotaxis test at 48 h (postnatal day 11, P11) postneonatal HI insult compared to control-treated animals (Figure 4). The Kruskal–Wallis test showed significant variation in the mean-time in seconds between treatment groups (*p* = 0.0151). Dunn’s multiple comparisons test showed significant decrease of the time necessary for change of orientation in the EV-treated compared to the HI-alone group (*p* = 0.0114) (Figure 4).

## Discussion

Our study shows evidence for neuroprotective effects of MSC-derived EVs via intranasal administration following neonatal HI brain injury, using the Rice–Vannucci mouse model in P9 mice. Intranasal treatment with MSC-derived EVs, immediately after severe (1 h) hypoxia following unilateral carotid artery occlusion, significantly reduced microglial activation, cell death, and tissue loss in the various brain regions tested, compared to brains in control-treated or untreated groups. In addition, compared to control littermates, intranasal EV treatment significantly improved short term behavioral outcomes as assessed through negative geotaxis.

Microglial activation was significantly reduced in cortex, hippocampus and striatum, while reduction in cell death was found in cortex and external capsule in the EV-treated group. Tissue volume loss was significantly reduced in pyriform cortex, thalamus and external capsule in the EV-treated group. Assessing all brain regions, the total brain regional means were also significantly decreased for microglial activation and tissue volume loss in the EV-treated groups.

These results are in line with findings from previous studies, showing differential vulnerability of certain brain regions to injury (Rocha-Ferreira and Hristova, 2016). HI injury at term, as modeled in this study, tends to damage the entire brain, most notably gray matter in cortex, hippocampus, and/or thalamus (Alexander et al., 2014).

Damage to the cortex, as well as to the thalamus and striatum (Lubics et al., 2005) have been associated with sensorimotor deficits in animal models of HI (Mercuri and Barnett, 2003; Mercuri et al., 2004; Martinez-Biarge et al., 2011). HI damage in the hippocampus and associated projections to the cortex have been shown to result in disrupted memory function and spatial processing (Aylward, 2005). Significant reductions in hippocampus volume have been shown to reduce long-term reference memory, short-term working memory (Ikeda et al., 2001) and impact the necessary role of the hippocampus in spatial navigation and recollection (Packard and McGaugh, 1996; White and McDonald, 2002). The dorsal striatum, namely the nucleus accumbens, may impact non-spatial navigation, and learning (Packard and Knowlton, 2002; White and McDonald, 2002), thus damage to this structure may account for the non-spatial memory deficits seen for example in HI injured rats (Alexander et al., 2014). Fronto-striato-thalamic circuitry damage from HI injury may lead to deficits in attention, executive function, and activity modulation (Aylward, 2005). As studies have shown anxiety-like behavior in mice following HI injury (Sanches et al., 2013), it has been hypothesized that HI injury may be associated with susceptibility to other pathologies such as attention-deficit hyperactivity disorder (ADHD), autism and schizophrenia, as the hippocampus and striatum are associated with related cognitive functions (DeLong, 1992; Lou, 1996; Dilenge et al., 2001; Van Petten, 2004; de Haan et al., 2006).

The results of the present study, emphasize the clinical potential of MSC-derived EVs for neuroprotection following neonatal HI injury and are in line with studies using MSC treatment in neonatal HI murine models (van Velthoven et al., 2010, 2012; Kim et al., 2012; Donega et al., 2014) as well as MSC-derived EVs treatment in an ovine neonatal HI model (Ophelders et al., 2016). The use of EVs as therapeutic vesicles is thus of great clinical interest. Previous HI studies using whole MSCs have found neuroprotective potential for these stem cells (van Velthoven et al., 2010; Donega et al., 2014; Park et al., 2015; Ahn et al., 2016; Corcelli et al., 2018) and the therapeutic time window was shown to be extended when combining MSC treatment with hypothermia (Ahn et al., 2018).

As there may be practical drawbacks of using whole MSCs in clinic, the use of MSC-derived EVs has been gaining increased interest. Neuroprotective effects were for example shown in a recent HI study following intravenous administration of MSC-derived EVs in an ovine model of transient umbilical cord occlusion *in utero*, with improved brain function and reduction of the number and duration of seizures following treatment (Ophelders et al., 2016). It has been shown that intravenous MSC delivery to the brain is hampered by the blood brain barrier (BBB) and results in a tendency of the MSCs to accumulate in other organs, such as the lungs (Lappalainen et al., 2008; Fischer et al., 2009). Intra-arterial administration can deliver high numbers of MSCs to the brain (Lappalainen et al., 2008; Li et al., 2010; Pendharkar et al., 2010), however, this method also has high incidences of mortality and impaired cerebral blood flow in rat HI models (Walczak et al., 2008; Pendharkar et al., 2010). Attempts to increase cell delivery by disrupting the BBB can leave the animal susceptible to infection or toxins (Burgess et al., 2011). Intranasal administration has shown to be superior to intravenous administration in experimental HI models due to direct transport to the CNS through intranasal drug delivery (Merkus and van den Berg, 2007) via the olfactory and trigeminal neural pathways, which innervate the nasal cavity and create a direct pathway to the CNS (Hanson and Frey, 2008). Thus in this study we delivered MSC-derived EVs intranasally, as this method is more efficient and far less invasive than intracranial, intravenous or intraarterial deliveries and avoids inactivation by the gastrointestinal and hepatic first-pass metabolism (Hanson et al., 2013; Djupesland et al., 2014; Kozlovskaya et al., 2014). The MSC-derived EVs used in this study are well characterized (Collino et al., 2015; Bruno et al., 2017; GEO GSE59958; Supplementary Figure S1) and have previously shown to regenerative potential in murine acute renal injury models (Bruno et al., 2012; Bruno et al., 2017).

In the current study, we assessed the neuroprotective effects of MSC-derived EVs following intranasal administration after a HI-insult. We observed significant neuroprotective effects and improved short-term behavioral outcomes. This bodes well for future applications of MSC-derived EVs, or EVs derived from other stem cells, following neonatal HI and may also be translatable to other types of neurotrauma. Tracing of MSC-derived EVs, using PKH26 (Long et al., 2017), would further provide information on the exact fate of intranasally delivered EVs to specific brain-regions and such tracing of EV fate is indeed planned by our group, alongside assessment on longer-term outcomes following MSC-EV treatment. The application of stem-cell derived EVs in combination with hypothermia, for an extended therapeutic time window, should also be explored. The preparation of the EVs is relatively inexpensive and simple, but for successful translation into the clinic strict quality control of MSC cultures and well characterized MSC-derived EVs will be required. Overall our data suggests that intranasal application of MSC-derived EVs following neonatal HI insult has neuroprotective effects and thus possesses potential as clinical treatment for neonatal HI brain damage.

## Data Availability

All data generated or analyzed during this study are included in this published article.

## Author Contributions

CS and MH carried out the animal experiments. SK, MHS, SB, MD, GC, and JI prepared and characterized the MSC-EVs. CS, JN, and MH generated the histological and behavioral data. All authors contributed to data analysis and read and approved the final manuscript. SL and MH co-designed the study and wrote the manuscript.

## Conflict of Interest Statement

The authors declare that the research was conducted in the absence of any commercial or financial relationships that could be construed as a potential conflict of interest.

## Supplementary Material

FIGURE S1 Characterization of the MSC-derived EVs used in the current study for intranasal application following HI. **(A)** Nanosight analysis showing EVs in the size range of 30–1000 nm. **(B)** Electron microscopy showing purified MSC-derived EVs, the scale bar represents 0.5 μm. **(C)** FACS analysis verifying the presence of the following MSC-EV specific markers: CD105, CD44, CD73, CD29, CD63, and CD81. **(D)** Western blotting showing MSC-EV specific markers CD63 and CD9.
