# Supplementary figures and images for: Mesenchymal Stromal Cell Derived Extracellular Vesicles Reduce Hypoxia-Ischaemia Induced Perinatal Brain Injury
Source: Front Physiol. 2019 Mar 19;10:282. doi: 10.3389/fphys.2019.00282 (PMC6433879; doi:10.3389/fphys.2019.00282)

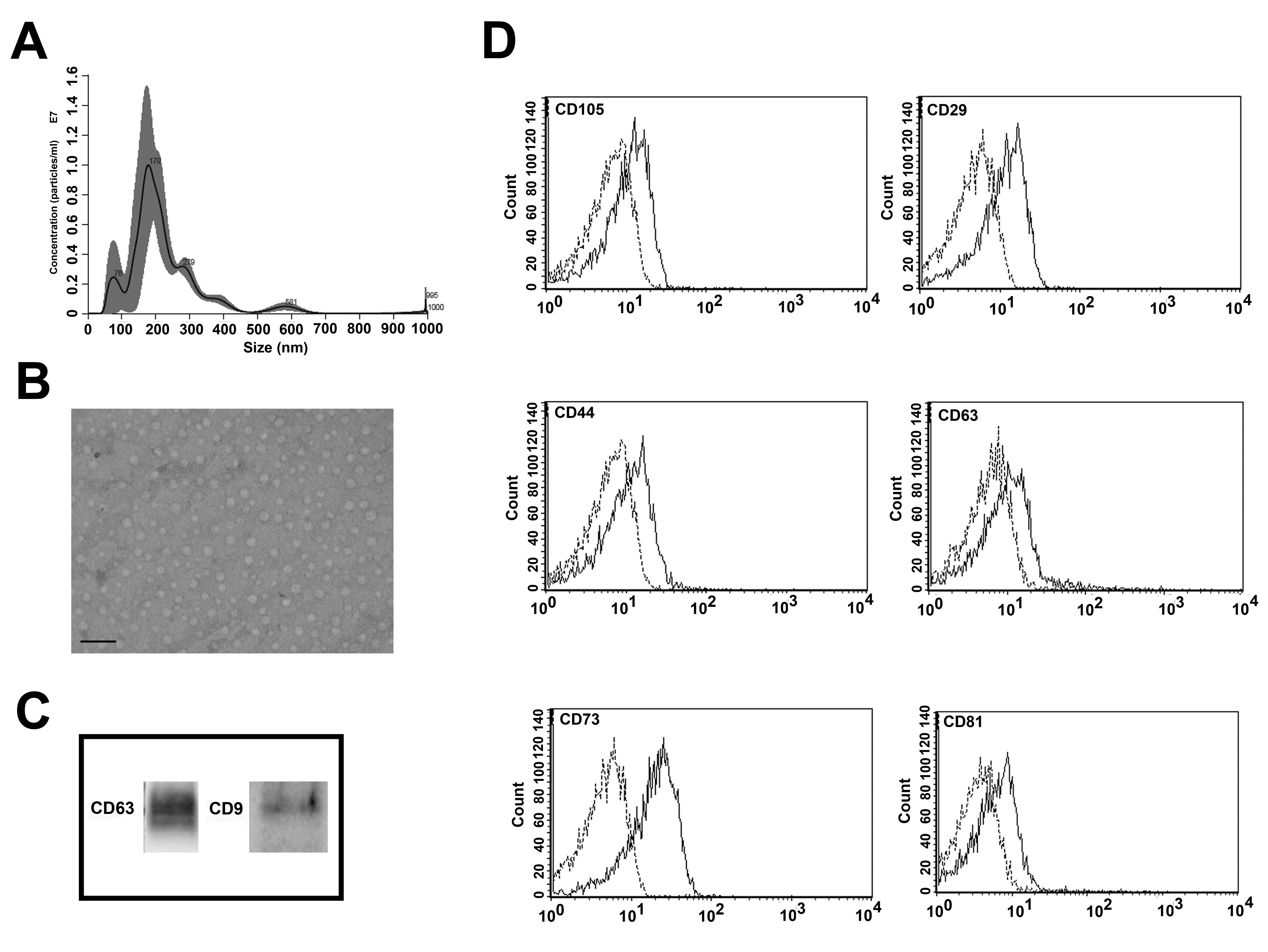

Supplement: FIGURE S1 — Characterization of the MSC-derived EVs used in the current study for intranasal application following HI. (A) Nanosight analysis showing EVs in the size range of 30–1000 nm. (B) Electron microscopy showing purified MSC-derived EVs, the scale bar represents 0.5 μm. (C) FACS analysis verifying the presence of the following MSC-EV specific markers: CD105, CD44, CD73, CD29, CD63, and CD81. (D) Western blotting showing MSC-EV specific markers CD63 and CD9. [file Image_1.TIF]
